# Collaborative study on the genetics of alcoholism: The strength of collaboration, team science, and longitudinal data

**DOI:** 10.1111/gbb.12866

**Published:** 2023-09-19

**Authors:** Marissa A. Ehringer

**Affiliations:** ^1^ Institute for Behavioral Genetics and the Department of Integrative Physiology University of Colorado Boulder Colorado USA

**Keywords:** alcohol, cell culture, collaboration, editorial, genetics, genomics, imagining, longitudinal, omics, team science

## Abstract

This issue contains a series of articles describing the various resources, studies, results, and future directions for the collaborative study on the genetics of alcoholism (COGA). The collaborative and integrative approach initiated by this group ~30 years ago serves as an excellent example of the strength of team science. Individually, various aspects of COGA would be limited in their impact toward improved understanding of alcohol use disorder. Collectively, their wholistic approach which spans deep longitudinal phenotypic assessments in families to include the application of large‐scale omics technologies and cell‐culture based molecular studies has demonstrated the power of working together.

Over the past 10–20 years, the importance of collaborative team science, especially as applied to the study of complex multigenic behavioral traits such as alcohol use disorders (AUDs), has become key to effectively moving the field forward. Human genome‐wide association studies have been successful at identifying genes associated with AUDs and other substance use and psychiatric disorders because of the dedication of researchers who have shared data across studies to achieve the sample sizes necessary to detect associations for SNPs contributing small effects toward risk for these diseases.

The collaborative study on the genetics of alcoholism (COGA), which was initiated in 1989 as a pioneering integrative effort across multiple sites in the United States. COGA brought together investigators across disciplines, all who share the goal of understanding both biological and environmental risk factors for AUDs and possible treatments. This issue includes five articles describing the work spanning three decades, which is broadly reviewed in four areas: sample and clinical data,^1^ brain function,^2^ genetics^3^ and functional genomics,^4^ with an introductory overview.^5^ The COGA study exemplifies elegance in its ability to apply creative ideas, innovative approaches, critical thinking and patience while working within a framework of collaborative science.

The original investigators recognized the value of a longitudinal family‐based study design where a rich collection of phenotypic measures was collected at regular intervals. As our knowledge of environmental factors, genetics and biology has evolved, they built upon the original infrastructure to recruit additional generations of subjects and to expand the diversity of the sample to include a substantial proportion of black and Hispanic subjects. This ancestral diversity is critically important for future studies, since there are known group differences due to both environmental and genetic factors. Though the overall sample size (*N* = 17,878) is “small” by today's GWAS standards, the richness of data is exceptional and likely to be critical for interpretation of GWAS polygenetic risk scores.

Furthermore, COGA is unique in that investigators expanded assessments in early stages of the study beyond standard questionnaires and DNA genotyping to include brain imaging data. Currently, functional data have been collected for nearly 10,000 participants, of which nearly 8000 have been assessed at more than one time point. Many of these measures were collected during cognitive assessments, providing novel insight about neuronal responses in “real time” while performing a task.

Functional genomics studies have employed human brain tissue to identify differences in gene expression patterns between subjects with and without AUD, and induced human pluripotent cells from COGA participants have been developed to study the effects of genetic variants on a variety of functional responses to alcohol. Importantly, these studies are closely integrated with discoveries from the other components of the larger study, taking into account possible environmental perturbations, underlying genetic risk scores and functional brain responses.

Finally, COGA has fostered and supported an environment of data‐sharing and mentoring. Multiple COGA investigators have participated in larger meta‐analyses of genetic data by contributing COGA data, which is available through NIAAA, dbGAP, or through direct collaboration. Many of the current leaders of COGA initially started working with the data as graduate students, and have progressed through their career as postdoctoral fellows to become full Professors and leaders in the field now training the next generation of scientists. As a team, they are well‐poised to lead the field as cutting‐edge approaches and high‐throughput technologies continue to emerge. Given the complexity of AUDs at the behavioral, environmental and physiological levels, this team science approach is the most promising strategy to advance novel therapies in a future of personalized medicine.

## FUNDING INFORMATION

MAE is supported by NIH U01 DA051937, R21 DA05781, and AB Nexus from the University of Colorado.
